# Structure Characteristics Affected by Material Plastic Flow in Twist Channel Angular Pressed Al/Cu Clad Composites

**DOI:** 10.3390/ma13184161

**Published:** 2020-09-18

**Authors:** Lenka Kunčická, Zuzana Klečková

**Affiliations:** 1 Institute of Physics of Materials, ASCR, 61662 Brno, Czech Republic; 2 Department of Thermal Engineering, Faculty of Metallurgy and Materials Engineering, VŠB TU Ostrava 17. Listopadu 15, 70833 Ostrava-Poruba, Czech Republic; zuzana.kleckova@vsb.cz

**Keywords:** clad composite, rotary swaging, finite element analysis, effective strain, residual stress

## Abstract

The study focuses on structure analyses, texture analyses in particular, of an Al/Cu clad composite manufactured by single and double pass of the twist channel angular pressing (TCAP) method. Microscopic analyses were supplemented with numerical predictions focused on the effective imposed strain and material plastic flow, and microhardness measurements. Both the TCAP passes imparted characteristic texture orientations to the reinforcing Cu wires, however, the individual preferential grains’ orientations throughout the composite differed and depended on the location of the particular wire within the Al sheath during extrusion, i.e., on the dominant acting strain path. The second TCAP pass resulted in texture homogenization; all the Cu wires finally exhibited dominant A fiber shear texture. This finding was in accordance with the homogenization of the imposed strain predicted after the second TCAP pass. The results also revealed that both the component metals exhibited significant deformation strengthening (which also caused bending of the ends of the Cu wires within the Al sheath after extrusion). The average microhardness of the Cu wires after the second pass reached up to 128 HV, while for the Al sheath the value was 86 HV.

## 1. Introduction

Composite materials can generally be characterized as materials consisting of two or more phases or components featuring different physical and chemical properties, separated by mutual interfaces [1,2]. The existence of the interfaces differentiates composites from alloys manufactured conventionally, e.g., by melting and subsequent casting. Combining different metals (featuring advantageous formability, strength, and thermal and electrical conductivities), possibly with other materials, such as ceramics (featuring favorable hardness, strength, tolerance to high temperatures, and low thermal expansion), brings about the possibility to produce composite materials with higher utility properties than those of numerous single-phase materials and alloys [3,4].

Quite a wide spectrum of composites has been presented so far and their characterization can be performed according to various criteria. Generally, composites consist of a matrix (i.e., first phase), and (several) other phase(s), typically added to enhance the mechanical properties of the final product (i.e., reinforcing phase(s)) [5]. Therefore, composites are usually characterized according to the type of the reinforcing phase. The first type is composites with continuous reinforcing elements, typically long fibers, i.e., continuous composites, whereas the second type is composites with discontinuous reinforcing elements, typically particles, whiskers, short fibers, etc., i.e., discontinuous composites. When compared to the first type, the second type is advantageous due to its easier and cheaper production and variability of the final shape of the product achievable during possible secondary treatment (forging, rolling, extrusion, severe plastic deformation (SPD) processing, etc.) [6].

Regardless of the type of composite, the grain size and distribution of the reinforcing phase within the composite have a major influence on the effectivity of enhancement of the mechanical properties. Similar to conventional materials, among the aims of the production of composite materials is to acquire homogeneous fine-grained structures with favorable distributions of the reinforcing elements within the matrices. However, simultaneous achievement of complete consolidation (high quality bonding of the phases) and an ultra-fine-grained (UFG) structure is a challenge when performed via conventional methods, such as hot isostatic pressing (HIP) or extrusion of powder-based materials, and forging of presintered semiproducts [7].

Among the promising methods for the production of UFG composites is powder metallurgy, which can advantageously be used to fabricate microcomposites and nanocomposites when using nanopowders (the size of the individual powder particles is up to hundreds of nanometers). This production method is applicable especially for tiny components for the electrotechnics [8,9]. The main advantage of such composites is negligible final porosity and high quality of bonding of the individual powder particles. Among the positive aspects of using nanopowders is also the possibility to apply lower pressures and temperatures during their consolidation/sintering [10], which enables the application of more or less conventional forming methods for the production of such materials. However, the works documenting achievement of the required structure refinement or desired redistribution of the reinforcing elements by conventional forming technologies are scarce (e.g., [11,12,13]). Nevertheless, compact composites with negligible porosity and a very fine structure can be fabricated via methods of intensive plastic deformation enabling significant structure refinement (formation of UFG structure) without introducing substantial changes in shapes of the processed billets. Intensive shear deformation imparted by the combination of high pressure and rotary movement processed at room/elevated temperatures also supports mutual bonding of the used materials [14,15,16,17]. The SPD technologies mostly used to prepare composite materials are the equal channel angular pressing (ECAP) [18,19,20] and ECAP-based methods [21,22], high pressure torsion (HPT) [23], and accumulative roll bonding (ARB) [24]. ECAP and HPT are advantageous for the consolidation of powders, as well as for their subsequent processing to the final shapes, whereas ARB is especially favorable for enhancing structure refinement of preconsolidated materials [25], or for the preparation of clad composites [26]; the latter has been successfully performed also via the repeated folding (RF) process, the fundamentals of which are similar to ARB [27].

Despite the fact that virtually all the SPD methods are applicable for the preparation of composites, the (sub)structure development is different for multi-phase materials, and single-phase ones. For single-phase materials deformed via methods such as ECAP, substructure formation involves increasing dislocation density and development of cell blocks with large misorientations resulting in the formation of dislocation cells the size of which typically decreases with increasing imposed strain. With continuing deformation, such a substructure gradually transforms into a homogeneous structure featuring fine grains with a high portion (up to 85%) of high angle grain boundaries [28]. The minimum achievable grain size for single-phase materials deformed by SPD methods is primarily controlled by the imposed strain, its character (single pass, cyclic, etc.), and processing temperature. On the other hand, the process of (sub)structure formation within multi-phase materials, i.e., composites, deformed via SPD methods is different. Generally, the decrease in the grain size is not directly proportional to the imposed strain; decreasing thickness of the reinforcing component supports a decrease in the grain size within this component during processing. However, the application of critical deformation (i.e., imposed strain, the value of which depends on the material) leads to the formation of amorphous structures and saturated solid solutions, regardless of the used technology [29,30,31,32]. Due to the interactions of the individual phases during intensive plastic processing of composites, the achievable grain size for them is generally smaller than for the original single-phase materials; SPD methods are applicable for the preparation of nanocomposites with the average grain sizes of 10 nm [33]. Therefore, the intensive plastic deformation is promising for the preparation of innovative multi-phase composites with enhanced properties.

A specific type of composite is clad composites (sometimes characterized as layered or hybrid materials) consisting of two or more different metals bond at mutual interfaces. These materials are gaining increasing interest in various industrial branches and have been subjects to numerous studies reporting the application of forming technologies for their preparation, e.g., Al/steel/Al, Ti/Cu, Ti/Al, and Mg/stainless steel were prepared using conventional rolling [34,35,36]. Nevertheless, rolling is usually combined with other processing technologies, as documented, e.g., by the study reporting the application of explosive bonding and subsequent rolling for the preparation of a Ti/steel clad composite, combining the excellent strength of steel and corrosion resistance of Ti, for the petrochemical industry [37]. Given by the positive effects of the different rotation rates of the rolls on the imposed shear strain, asymmetrical rolling was used to prepare clad composites combining Al and Cu [38,39].

The combination of Al and Cu is specifically favorable since both the metallic components exhibit excellent electric and heat conductivities. Replacing a portion of Cu by Al results in a material featuring lighter weight and reduced cost when compared to Cu. Optimized processing of the composites provides advantageous materials applicable, e.g., in the aerospace and automotive [40,41], or electrotechnics [42]. Given by the combination of light weight and favorable mechanical properties, Al/Cu composites are believed to possibly supplement/replace steel in particular applications in vehicles and aircrafts [41]. The studies documented favorable durability of the Al/Cu clad composites during dynamic loading (strain rates up to 100 s^−1^), which can primarily be attributed to the development of deformation induced twins. The intensive shear strain does not only support grain refinement, but also introduces friction at mutual interfaces (given by different plastic flows of the component metals), which consequently generates heat supporting mutual bonding of the metals via atomic diffusion. Nevertheless, increasing temperature can also result in the formation of interlayers (intermetallics), which can develop within both, the continuous and discontinuous composites.

Since the conventional forming methods and heat treatments feature certain limitations, unconventional forming, i.e., via the SPD methods, has become favorable also for the preparation of clad composites. SPD is beneficial especially from the viewpoint of introducing homogeneous distribution of secondary particles, as well as grain refinement and possibly UFG structures within the composite metals. Moreover, the shear strain supports corrupting of possible oxide layers at the surfaces of the component metals and increases diffusion via introducing lattice defects [43,44]. Numerous studies have documented substantial enhancement of the final properties of clad composites when prepared using SPD [16,17,45,46,47]. Probably the most promising is the HPT method, which successfully suppresses the development of cracks via applying very high pressures. It also imposes very high strains, which supports redistribution of secondary particles. For the conventional ECAP technology, the strain imposed during a single pass can be increased via modifications of the dies, or by implementing back pressure.

The twist channel angular pressing (TCAP) method combines twist and bend deformation zones within a single equal channel, which enables to impart a substantial shear strain during a single pass [48,49]. TCAP has been successfully proven to impart homogeneous UFG structures, and the effectivity of single pass TCAP has been documented to be higher than double pass ECAP [22,50,51].

Since TCAP has only been used for single-phase materials so far, this study focuses on the characterization of the grains’ orientations and plastic flow within an Al/Cu clad composite processed via single and double pass TCAP. Experimental texture analyses are supplemented with numerical simulations predicting the effective imposed strain values and distribution throughout the extruded composites, as well as documenting the behavior of the billet during processing, especially the behavior of the reinforcing Cu wires within the Al sheath. Last but not least, mapping of microhardness throughout the extruded billets’ cross-sections was performed.

## 2. Materials and Methods

**Figure 1:**
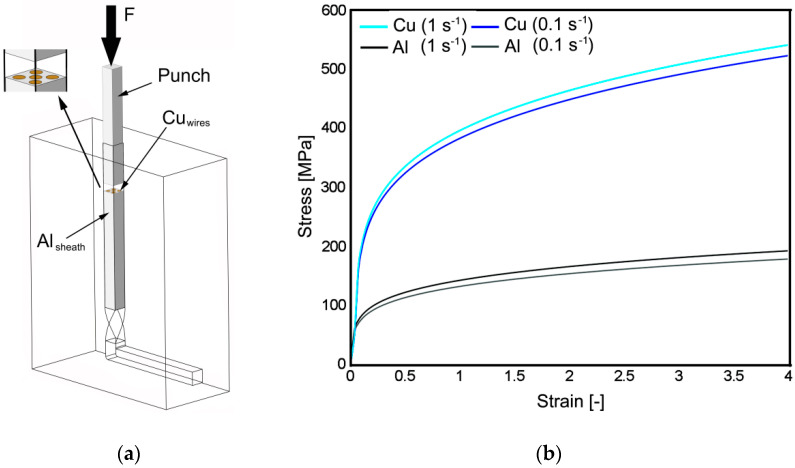
Schematic depiction of twist channel angular pressing (TCAP) processing of the Al/Cu clad composite (**a**) and experimental stress–strain data used for numerical simulations (**b**).

The aim of the presented work was to perform detailed characterization of the influences of the applied TCAP process on the behavior of grains within an Al/Cu clad composite reinforced with Cu wires. The study primarily involves the experimental double-pass extrusion, and is supplemented with the numerical prediction of the materials’ behaviors. Schematics of the TCAP process acquired from the numerical simulation can be seen in Figure 1. The clad composite consisted of an Al sheath and five reinforcing Cu wires. Both the experimental and numerically simulated billets were processed via a single pass TCAP, and subsequently via the second pass, deformation route *A* for which was selected (i.e., the billet was not rotated between the individual passes—details of the selected deformation route and strain paths can be found in [52,53]).

The first part of the study was focused on the practical realization of the TCAP process. The selected component metals were commercially pure (CP) Cu (99.97%) (Ferona, a.s., Prague, Czech Republic) with the composition of (in wt %): 0.0074 Ni, 0.0058 Sn, 0.0031 Fe, 0.0030 Zn, 0.0023 Si, and bal. Cu; and CP Al (99.97%) with the chemical composition of (in wt %): 0.125Fe, 0.020Cu, 0.020Zn, 0.015Mg, 0.015Mn, 0.015Ti, 0.10Si, and bal. Al. Before preparing the composite billet, all the components were annealed at 500 °C for 30 min in an electric furnace. The dimensions of the initial composite billets were 12 mm × 12 mm (square cross-section) × 130 mm (length). The processing was carried out at room temperature using a hydraulic press, MoS_2_ (Ferona, a.s., Prague, Czech Republic) was applied as the lubricant. The extrusion rate was 5 mm s^−1^.

The texture analyses of the clad composites processed via the single and double TCAP pass were performed via the SEM-EBSD method (scanning electron microscopy-electron backscatter diffraction). The samples for the analyses were prepared by manual grinding on SiC papers and subsequent manual/electrolytic polishing by a specific procedure developed by Dr. Michal Jambor, Institute of Physics of Materials, CAS. SEM-EBSD analyses were done using a Tescan Lyra 3 FIB/SEM microscope equipped with a NordlysNano EBSD detector (Oxford Instruments, Abingdon-on-Thames, Great Britain). EBSD scanning was carried out with the accelerating voltage of 20 kV and scan steps of 50 nm. The structure analyses and texture evaluations were performed using AZtecCrystal 1.1 software, and ATEX software [54]. The mechanical properties of both the extruded composites were investigated by microhardness measurements on cross-sectional cuts from the billets (perpendicular to extrusion axis) using a Zwick/Roell testing machine. The indents spacing was 1 mm and the applied parameters per indent were: load of 200 g and loading time of 10 s. The maps were assembled by the own script programmed by Adam Weiser, Institute of Physics of Materials, CAS.

**Figure 2:**
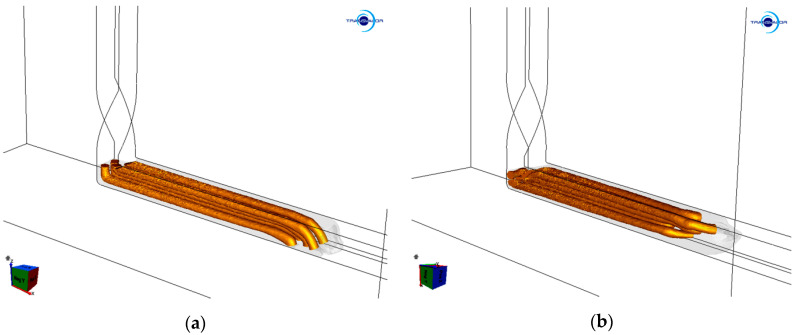
Deformation behavior of the extruded billet during: single pass (**a**) and double pass (**b**).

The experimental study was supplemented with numerical simulations of the single and double pass extrusions. The behavior of the composite billet during single and double pass was predicted using the Forge NxT commercial software by Transvalor implementing the finite element method (FEM). The geometrical dimensions and mechanical properties in the numerical model corresponded to the real experimental parameters. The friction was determined as the Coulomb friction (µ = 0.02) and the boundary conditions specified in Table 1 were defined on the basis of a previously performed study in which they were experimentally validated [52]. The geometry of the TCAP die used in both the simulation and experiment was defined by the following angles: ω = 90°, φ = 90°, β = 40°, and ψ = 20° (twist rotation angle, channel bending angle, twist slope angle, and arc of curvature of channels’ intersection angle, respectively—the angles are described in detail, e.g., in [48,49]). The component metals were characterized using an elastic–plastic model defined by the Newton–Raphson convergent algorithm, and the die and extruder were defined as rigid parts. The clad composite billet was meshed with tetrahedral elements (215, 871 nodes in total), and automatic remeshing was activated since intensive shear deformations were expected to proceed. The room temperature stress–strain data at the strain rates of 0.1 and 1 s^−1^ (see Figure 2) imported into the database of the computational software was acquired experimentally for both the used composite metals via torsion tests performed using a servo-hydraulic torsion plastometer (SETARAM). Material behavior was finally determined via the Hansel–Spittel equation (Equation (1)),

(1) $$\sigma_{f}=Ae^{m_{1}T}T^{m_{8}}\varepsilon^{m_{2}}e^{m_{4}/\varepsilon }{\left(1+\varepsilon \right)}^{m_{5}T}e^{m_{6}\varepsilon }{\dot{\varepsilon }}^{m_{3}}{\dot{\varepsilon }}^{m_{7}T},$$

where *ε* is the equivalent strain, *T* is the temperature, $\dot{\varepsilon }$ is the equivalent strain rate, and *A*, *m*_1_, *m*_2_, *m*_3_, *m*_4_, *m*_5_, *m*_6_, *m*_7_, *m*_8_, and *m*_9_ are regression coefficients. The values of the coefficients for Cu were 411.19 MPa, −0.00121, 0.21554, 0.01472, and −0.00935, respectively, and *m*_5_ ÷ *m*_8_ was 0. The values of the coefficients for Al were 151.323 MPa, −0.00253, 0.21142, 0.03177, and −0.00654, respectively, and *m*_5_ ÷ *m*_8_ was 0.

## 3. Results

### 3.1. Numerically Predicted Plastic Flow and Imposed Strain

The results of the numerical simulation showed that the billets extruded via single and double pass TCAP exhibited differences in their deformation behaviors, as did both the component metals. Figure 2a,b shows images from the simulations, the billets extruded via one and two TCAP passes in which were depicted, respectively. In Figure 2a,b, the Al sheath was visible as a contour so that the shapes of the Cu wires could clearly be seen. Both the TCAP passes imparted significant plastic deformation of both the composite components. However, differences between the single and double pass were observed, especially for the Cu wires.

As can be seen from the prediction, the ends of the Cu wires were bent downwards to the bottom of the horizontal channel after the single pass (Figure 2a), which originated when passing through the channel bending, i.e., the main deformation zone (MDZ). Additionally, the two upper wires were shifted forward after extrusion. This phenomenon can be attributed to the fact that, in the MDZ, the material plastic flow in the upper cross-sectional region of the composite billet was faster than in its bottom cross-sectional region. Additionally, the shear strain imposed to the composite in the MDZ was more significant along the inner periphery of the channel bending than along its outer periphery [55].

After the simulated double pass, the Cu wires were bent towards the side of the horizontal channel (Figure 2b). This can primarily be attributed to the selected deformation route (*A*, see Section 2 and references. [52,53]), which involved no rotation between the passes. By the effect of the presence of the twist deformation zone (TDZ), the locations of the four peripheral Cu wires changed during the second extrusion, i.e., the billet cross-section rotated by 90° compared to the cross-section of the billet extruded via a single pass. In other words, the Cu wire located in the upper left corner of the composite after the first pass shifted to the bottom left corner after the second pass, etc.

**Figure 3:**
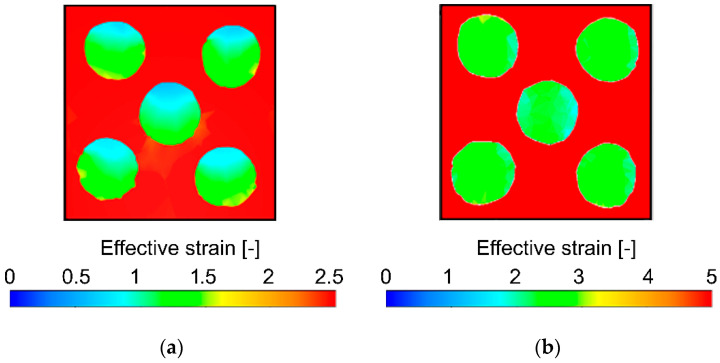
Effective strain distribution after: single pass (**a**) and double pass (**b**).

The development of the imposed effective strain during a TCAP pass is rather complicated. The TDZ primarily affects the peripheral/corner regions of the extruded billet, by the effect of which the imposed strain did not influence the reinforcing Cu wires as much as the (periphery of) Al sheath in this deformation zone. The Cu wires were not significantly affected by the shear strain during passing through the TDZ, but later on, during passing through the MDZ. As depicted in the cross-sectional cut through the simulated billet extruded via a single pass in Figure 3a, the Al sheath exhibited homogeneous imposed effective strain distribution of rather high values (up to 2.5) when compared to the Cu wires, the maximum effective strain values in which reached up to 1.5. Moreover, the imposed strain distribution through the wires’ cross-sections was not homogeneous after single pass TCAP. Both phenomena were caused by the significantly lower effect of the TDZ on the wires (compared to the Al sheath).

The prediction showed that after the double pass, the effect of the TDZ on the imposed strain was more evident than after the single pass for both the composite metals. As can be seen in Figure 3b depicting a cross-sectional cut through the billet extruded via double pass TCAP, the Al sheath exhibited the homogeneous distribution of the effective imposed strain (similar to the single pass), however, the maximum imposed strain values almost doubled (up to 5). The Cu wires exhibited not only an increase in the maximum imposed strain after the second pass, but also substantial homogenization of its distribution (please note the different scales, although the color scheme is maintained for both the Figure 3a,b—decreasing the range of the scale in Figure 3a enables better depiction of the inhomogeneities of the effective imposed strain within the Cu wires observed after the first TCAP pass).

### 3.2. Texture Characteristics of Extruded Composites

The effects of the TDZ and MDZ on texture developments within the experimentally extruded composite billets were evaluated via pole figures (PF), and via determination of the intensities of dominant ideal shear texture orientations [56,57,58]. Since the effects of the TCAP process on texture development within the Al sheath were comparable to its effects on texture development within a CP Al billet, which were characterized in the previously published study [50], this study primarily focuses on the Cu wires.

**Figure 4:**
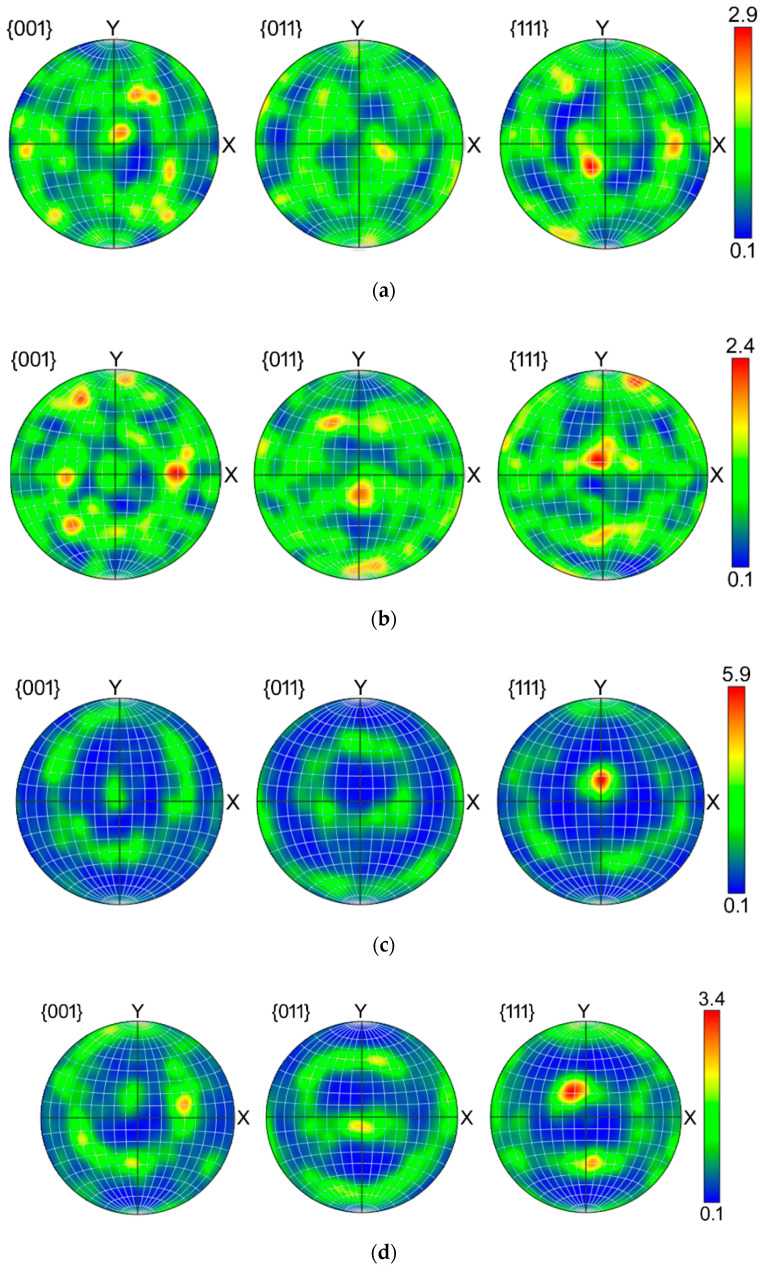

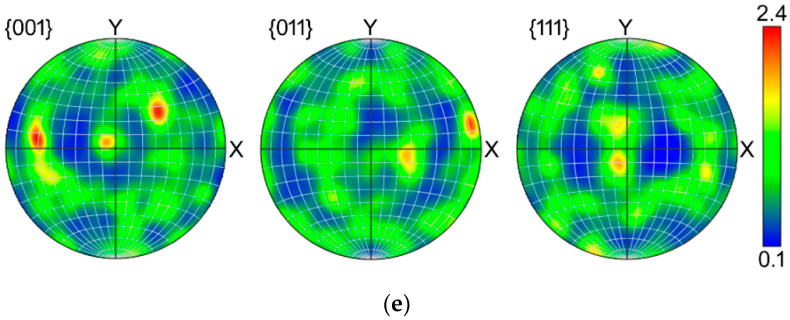
Pole figures for individual wires within composite billet extruded via single pass TCAP: upper left (**a**); upper right (**b**); axial (**c**); bottom left (**d**); and bottom right (**e**).

The original preannealed Cu exhibited more or less random texture with a slight tendency to form ideal cube recrystallization texture (not shown here). The texture orientations within the individual wires exhibited differences after the first pass—the individual {001}, {011}, and {111} PFs for the wires in the upper left, upper right, axial, bottom left, and bottom right positions are depicted in Figure 4a–e, respectively. As can be seen, the highest texture intensity was detected in the axial wire (Figure 4c), which exhibited dominant A fiber ideal shear texture orientation (i.e., {111}||shear plane) [56]. Regarding the peripheral wires, the pairs of the wires positioned across the cross-sectional diagonals exhibited tendencies to mirror. The bottom left (Figure 4d) and upper right (Figure 4b) wires primarily formed the C fiber ideal shear texture orientation (although by approximately 10° shifted), sometimes also denoted as the dominant α fiber defined by the Euler angles φ_1_, ϕ, and φ_2_ of 90°, 45°, and 0° [57,58], and the upper left (Figure 4a) and bottom right (Figure 4e) wires exhibited the tendencies to preferably form the Ab shear texture orientation (belonging to the A fiber, defined by the Euler angles φ_1_, ϕ, and φ_2_ of 180°, 35.26°, and 45°, respectively). However, the Ab ideal orientation was shifted towards the Bb ideal orientation (Euler angles φ_1_, ϕ, and φ_2_ of 180°, 54.74°, and 45°), especially within the upper left wire (Figure 4a).

**Figure 5:**
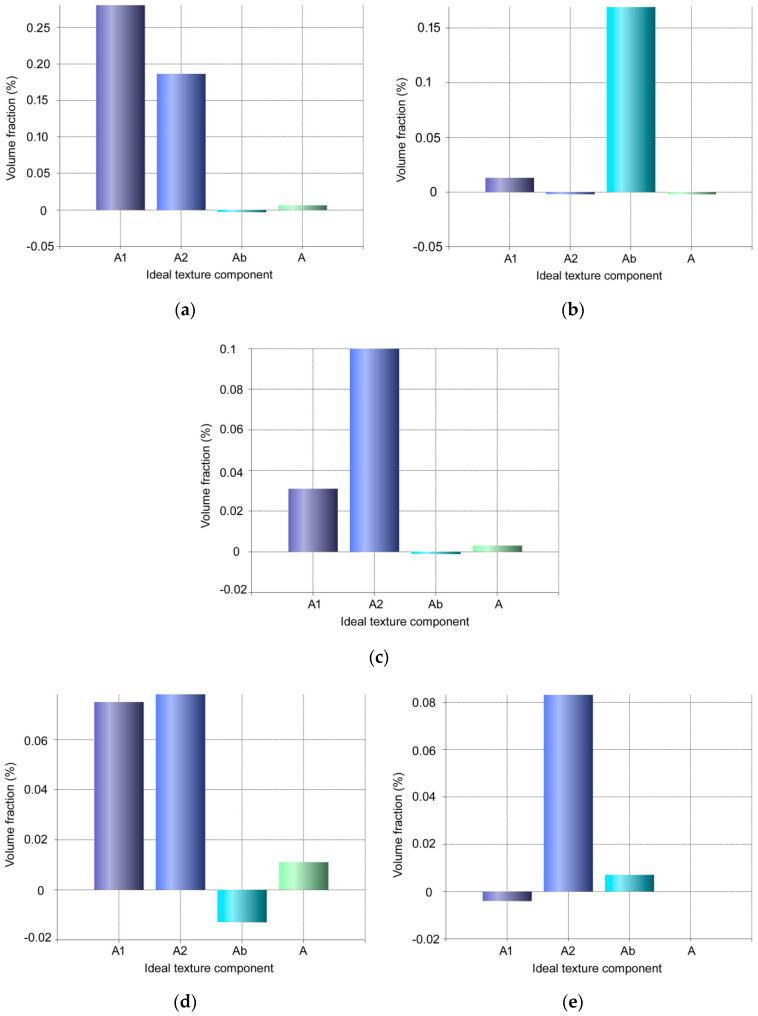
Volume fractions of dominant ideal texture orientations for individual wires within composite billet extruded via double pass TCAP: upper left (**a**); upper right (**b**); axial (**c**); bottom left (**d**); and bottom right (**e**).

After the experimental double pass TCAP, homogenization of the textures within the wires occurred. Figure 5a–e depicts the distributions of the main ideal texture orientations within the individual wires after the second TCAP pass. All the wires exhibited dominant texture orientations belonging to the A fiber, however, the suborientations varied slightly (orientations belonging to the B and C fibers are not depicted since their volume fractions were neglectable). A1 and A2 ideal texture components (Euler angles φ_1_, ϕ, and φ_2_ of 35.26°, 45°, and 0°; and 144.74°, 45°, and 0°, respectively) were dominant in all the wires after the second TCAP pass, except the upper right wire (Figure 5b), Ab ideal orientation for which was dominant. This wire was located in the bottom right position after the first pass (after which the texture already exhibited the tendency to form the Ab ideal orientation, see Figure 4e).

### 3.3. Microhardness and Structure of Extruded Composites

**Figure 6:**
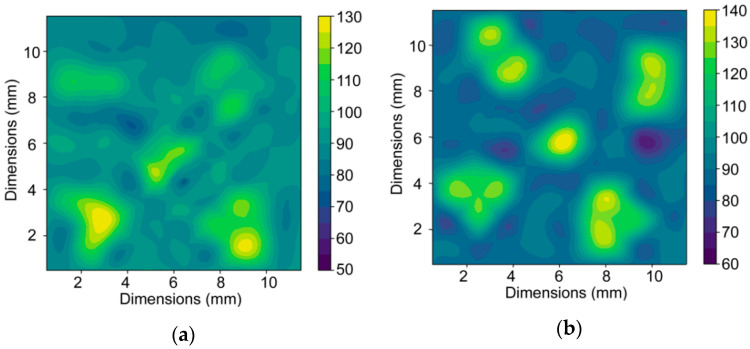
Maps of experimentally measured microhardness across a cross-section of billet after: single pass (**a**) and double pass (**b**).

The deformation strengthening introduced by the intensive shear strain and manifesting itself in macrodeformation and bending of the ends of the Cu wires (Section 3.1) contributed to increases in microhardness for both the experimentally extruded composite components. The original HV values of the initial annealed components were 58.6 for the Cu wires, and 37.4 for the Al sheath. Figure 6a,b depicts the maps of microhardness measured across the cross-sections of both the extruded billets. As can be seen, the microhardness values increased after both the single and double pass TCAP. After single pass TCAP, the average microhardness value of the Al sheath increased to 85 HV, and for the Cu wires it increased up to 107 HV. After the second pass, the average value of the Al was comparable to the first pass (86 HV), while for the Cu wires the average HV value increased to 128 HV.

**Figure 7:**
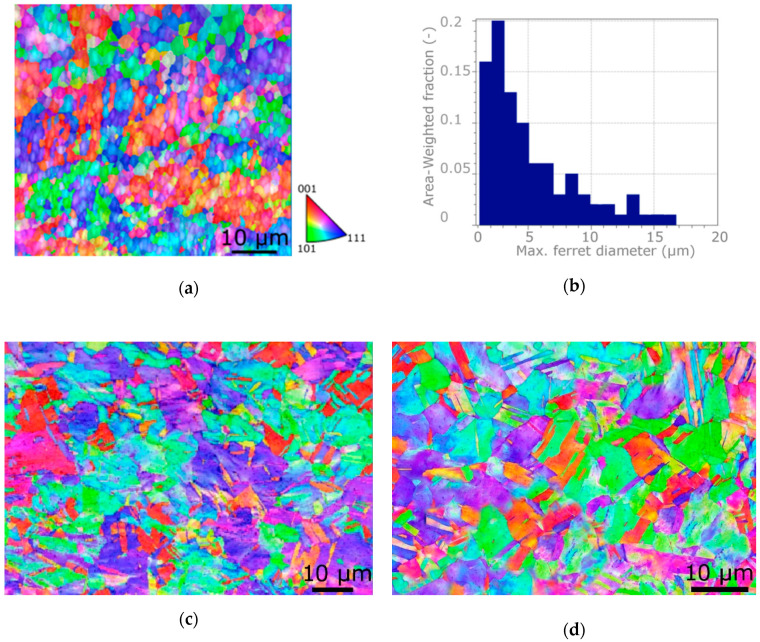
Structure of Al sheath after double pass TCAP: orientation image map (OIM) (**a**); grain size distribution (**b**); OIM of axial Cu wire after: Single TCAP (**c**); and double TCAP (**d**).

The results point to the fact that the Cu was not strengthened to its limit after the single pass, i.e., its structure was not saturated [59], by the effect of which the microhardness still increased after the second pass. On the other hand, the Al sheath featured structure saturation already after the first pass, and then exhibited structure recovery/recrystallization at the expense of further strengthening after the second pass. This supposition is in accordance with the grain refinement of the Al sheath occurring after both the passes. After single TCAP pass, the average grain size within the Al sheath was 5.4 µm, which was comparable with the values acquired during the previous study on a CP Al billet [50,51]. However, after the second pass, the average grain size within the Al sheath decreased down to 1.75 µm (see the orientation image map—OIM—and grain size distribution in Figure 7a,b). For comparison, the OIMs for the axial wire within the billets extruded via the single and double TCAP pass are depicted in Figure 7c,d, respectively, clearly showing that the grain size of the Cu wires was larger than within the Al sheath.

## 4. Discussion

Both the predicted and experimental results revealed that the deformation behavior of the composite billet was affected by the fact that it consisted of a combination of two different component metals. However, the individual deformation zones implemented within the die and the selected deformation route had non-negligible effects on the behavior of the billet, too. Passing through the TDZ did not generally introduce as high strains as passing through the MDZ [49]; the MDZ affects primarily the composite axial region, while the TDZ imposes high effective strain primarily to the composite (sub)surface regions. The TDZ thus imposed the shear strain primarily to the Al sheath, the plastic flow in the outer region of which was quicker than in the Cu wires. Subsequently, the MDZ contributed to the increase in the imposed shear strain also within the wires. The predicted bendings of the ends of the Cu wires after both the TCAP passes originating from different plastic flows of the individual regions of the composite billet developed during passing through the MDZ, where the upper cross-sectional region of the Al sheath adjoining to the Cu wires exhibited a tendency to flow quicker than the bottom cross-sectional region of the billet.

The development of texture during TCAP was affected by the acting strain path along which the shear strain was imposed [50,51]. As described above, the TCAP die is characterized by two independent deformation zones, twist zone (analogy to twist extrusion) two independent intersection planes in which are active, and bending zone (analogy to ECAP) another intersection plane in which is active. The TCAP die thus introduces severe shear strain into the extruded composite along three independent intersection planes, which also introduces a high amount of lattice distortions acting as obstacles for the movement of dislocations, and generation of nucleation sites during substructure formation (both these occurring phenomena were confirmed via the observed microhardness increase, and substantial grain refinement, see Figure 6 and Figure 7). After the first TCAP pass, the axial wire exhibited dominant A fiber texture orientation, which corresponds with the supposition of the minor effect of the TDZ on this wire during the first pass, since the A fiber is an ideal shear texture orientation developing during conventional ECAP. In other words, this finding documents the dominant effect of MDZ on this wire. The texture orientations identified in the peripheral wires were primarily affected by the two intersection planes along which the shear strain was imposed to the composite in the TDZ, especially as regards their tendency to mirror across the cross-sectional diagonals. Nevertheless, double pass TCAP resulted in homogenization of texture, as all the wires exhibited the tendencies to form A fiber dominant shear texture orientation.

## 5. Conclusions

The study presented the results of experimental analyses, supplemented with FEM numerical prediction, of an Al/Cu clad composite prepared by room temperature extrusion via the twist channel angular pressing (TCAP) method (single and double pass). The numerical prediction showed that both the passes introduced severe shear strain to both the component metals; the maximum effective imposed strain within the Al sheath of the billet processed via the double pass reached to the value of 5. The severe imposed strain subsequently introduced significant grain refinement and deformation strengthening (especially after the second pass) resulting in the increase in microhardness up to 128 HV for the Cu wires. The results also documented that the second pass introduced homogenization of the distribution of the imposed strain within the Al sheath and Cu wires, as well as uniform tendencies to form preferential texture orientations. The Cu wires exhibited the tendency to form A fiber preferential texture orientation after the second pass, although after the first pass, one pair of the peripheral wires exhibited the formation of A fiber texture, while the second pair formed C fiber texture.

## Figures and Tables

**Table 1:** Defined boundary conditions for component metals.

Property	Unit	Al	Cu
Temperature	°C	25	25
Young’s modulus	GPa	72	111
Poisson coefficient	-	0.3	0.3
Thermal expansion	K^−1^	24 × 10^−5^	1.7 × 10^−5^
Thermal conductivity	(W/(m.K))	250	394
Specific heat	(J.kg^−1^.K^−1^)	1230	398
Emissivity	-	0.03	0.7
Density	(g.cm^−3^)	2.80	8.96
